# Prescription Departmant

**Published:** 1892-07

**Authors:** 


PRURITIS ANI AND VULVÆ.—

The following formula will afford relief from the itching and irritation —to be applied locally:

R. Sodii Hyposulphis, dr. i
Acid Carbol. dr. ss
Glycerinæ, oz. i
Listerine, oz. iii M.

DIARRHŒA OF INDIGESTION, ETC.—

By *Prof. O. S. Armstrong*, Detroit, Mich.

R. Bismuthi subnitratis. dr. j.
Dyspepsinæ (Morse), scruple ii.
M. et. ft. chart. No. xii
Sig. (Small doses but effectual).

IN TYPHOID, a good formula is as follows:

R. Antikamnia, dr. ij
Ess. Pepsini.
Spts. Vin. Gallici, aa f oz. iv.

M. Sig.—Tablespoonful every three or four hours.

IRRITATIVE DYSPEPSIA WITH CONSTIPATION.—

Stearns' Cascara Aromatic, 1 fl. oz.
Fluid Extract Mandrake, 3 fl. dr.
Fluid Extract Senna, ½ fl. oz.
Fluid Extract Belladonna Root, ½ fl. dr.
Simple Syrup, q. s. 4 fl. oz.

Mix. Sig.: Dose, 1 to 2 teaspoonfuls.

This is of use in irritative dyspepsia, attended with constipation. It is particularly valuable from its modifying effects on the secretions of the alimentary canal.

SPASMODIC ASTHMA.—

R. Amm. iodide, dr. ij.
Amm. bromide, dr. iij.
Tinct. lobelia, oz. j.
Syrup tolu., oz iij.

M. Teaspoonful dose every one, two or three hours.

TYPHOID FEVER.—

R. Acid. carbolic, dram ss
Chloroform, ounce j
Gum acacia, dram j
Syrup, q. s. ounces iv

M. Sig.—Teaspoonful three or four times daily. If there is a tympanitic condition of the bowels, it disappears at once.—[I. J. Bush, M. D., *in Med. Ann.*

IMPOTENCE.—

Dr. W. F. Glenn claims ("Southern Practitioner") excellent results in impotence from:

R. Gold and sodium chloride, gr. iii
Strych. sulph., gr. i
Zinc phosphate., gr. iii
Ext. damiana, dr. i

M. and put into 30 capsules. Sig. One three times a day.

CHOLERA INFANTUM—Holt.

R. Naphthalini, grains xx to lxx

Ol. bergamii, gtt i-ij

M. Ft. chart, No xii.

Sig.—One powder every two or three hours.

TREATMENT OF BALDNESS.—

Whitla advises the following in baldness ("Med. News"):

R. Pilocarpin hydrochl. gr. v.

Otto rosæ, m. viii

Ol. rosmar., dr. iv

Lin. canthar., dr. iv

Glycerin pur., dr. viii

Ol. amygdal. dulc., dr. xvi

Spts. camphor, dr. xxiv

M. S. Rub well into the scalp night and morning.

INFANTILE DIARRHŒA.

R. Aq. calcis, ounces vj
Syr. rhei. arom , ounce j
Tr. opii. camph., ounce ss
Tr. Cardem. co., ounce ss

M. One teaspoonful often to children under one or two years, afflicted with sour stomach attended with diarrhœa, vomiting, etc.—*Memphis Med. Monthly.*

